# Prevalence of hypothyroidism among patients undergoing thyroid function tests in primary healthcare centres in Dubai, UAE

**DOI:** 10.1186/s12889-025-24355-y

**Published:** 2025-09-25

**Authors:** Sumaeya Abdulla, Budoor Alemadi, Latifa bin Shafar, Maryam Alsaeed

**Affiliations:** 1 Department of Family Medicine, Dubai Health, Dubai, UAE; 2 Department of Endocrinology, Dubai Hospital, Dubai Health, Dubai, UAE; 3 College of Medicine, Mohammed Bin Rashid University of Medicine and Health Sciences, Dubai Health, Dubai, UAE

**Keywords:** Autoimmune disease, Arab, Hypothyroidism, Middle East, Obesity, Prevalence, Primary healthcare

## Abstract

**Background:**

Hypothyroidism is a common disorder worldwide with a varied prevalence. Limited data are available on the prevalence rates of hypothyroidism in the Middle East and North Africa (MENA) region, particularly the United Arab Emirates (UAE). This study aimed to evaluate the prevalence and associated factors of hypothyroidism among adult patients visiting primary healthcare centres (PHC) in Dubai, UAE.

**Methods:**

A retrospective study was conducted using data from a registry maintained at the PHC of Dubai Health, from January 1, 2018, to December 31, 2020. The study included adults with thyroid function test (TFT) results and an International Classification of Diseases (ICD) 10 codes indicating hypothyroidism or subclinical hypothyroidism. Descriptive statistics were analysed for continuous and categorical variables, such as age, body mass index (BMI) and overweight or obese status. Chi-square and t-tests were employed to identify associations and differences between variables among individuals with and without hypothyroidism. The *p*-value ≤ 0.05 was considered statistically significant.

**Results:**

A total of 77,758 adult patients was analysed; among them, 49,995 (64.3%) were females and 27,762 (35.7%) were males; data was missing for 1 patient. The median age was 42 years, with a median BMI of 28.41. The overall prevalence of hypothyroidism was 2.1%, consisting of 91.4% overt hypothyroidism and 8.6% subclinical hypothyroidism. It was significantly higher in females (81.5%) than in males (18.5%). The highest prevalence (2.52%) was found in adults aged 35–44 years. Nearly half of the patients with hypothyroidism were obese (47%), followed by patients who were overweight (33.9%). Among patients with hypothyroidism, 0.73% had type 1 diabetes mellitus and no co-diagnoses of other autoimmune diseases; comorbidities included dyslipidemia (8%), hypertension (6.5%) and ischemic heart disease (1%).

**Conclusion:**

The study revealed that hypothyroidism is common among patients visiting PHC in Dubai, with a prevalence rate similar to those reported in Europe and North America (0.3%-5.3%), but much lower than that in neighbouring Gulf countries, such as Bahrain (44%), Saudi Arabia (29%) and Jordan (26%). Moreover, the highest prevalence of hypothyroidism is found among young and middle-aged individuals, particularly women.

## Background

Hypothyroidism is a common endocrine condition characterised by a deficiency in thyroid hormone production [1]. Most cases of hypothyroidism stem from primary thyroid disease related to autoimmune thyroiditis or other forms of inflammation, iodine deficiency, or congenital and iatrogenic causes [2]. Central hypothyroidism, secondary to pituitary disease, represents a less common cause. Due to the variability and non-specificity of symptoms, the definition of hypothyroidism primarily relies on biochemical indicators. Therefore, the diagnosis of hypothyroidism is based on biochemical testing with specific cut-off values for thyroid-stimulating hormone (TSH) released from the pituitary gland and free T4 (FT4) or thyroxine, free T3 (FT3), or triiodothyronine from the thyroid gland. Overt or clinical hypothyroidism is defined by an elevated concentration of TSH above the reference range and reduced levels of FT4 and FT3. Subclinical hypothyroidism is characterised by elevated TSH levels with normal FT4 and FT3 levels [3].

The global prevalence of hypothyroidism varies, with limited data in the UAE or the Gulf region. Apart from autoimmune disease and genetic factors, the major cause of hypothyroidism worldwide is iodine deficiency. As this differs across geographical locations and with national policies on iodine fortification of table salt, it is not surprising that the incidence rates vary among regions [4, 5]. In its most recent report, the Iodine Global Network identified eight countries in the Middle East and North Africa (MENA) region with < 70% of iodised salt coverage, with Iraq being classified as a country having iodine insufficiency [5]. In contrast, all countries in the Gulf Cooperation Council (GCC), which includes Saudi Arabia, Kuwait, Qatar, Bahrain, the UAE and Oman, have safe levels of iodine coverage [6]. Iodination programs have changed goitre endemicity rates, with significant variability observed historically in the MENA region and the UAE [7, 8].

Prevalence rates vary depending on the definition or reference ranges used for hypothyroidism. Older individuals tend to have a higher baseline TSH level [9–11] and females are significantly more susceptible to hypothyroidism than males [12, 13]. Also, the low threshold for testing, as well as generalised TSH cut-off values for the population, has led to an increase in the detection of subclinical hypothyroidism, which may have otherwise been asymptomatic [14, 15]. These variables have been explored extensively in large epidemiological studies on the prevalence of overt and subclinical hypothyroidism in Europe and the United States [13, 16–19].

The primary objective of this study is to determine the prevalence of hypothyroidism among patients in primary healthcare centres (PHCs) in Dubai, UAE. Secondary objectives include analysing the prevalence according to demographic groupings and exploring underlying factors for hypothyroidism in this patient cohort.

## Methods

### Study design

A retrospective study was conducted at the PHC of Dubai Health, a government-operated health service in Dubai, UAE. The study estimated the prevalence of hypothyroidism among patients who underwent TFTs within the PHC, obtained by following the International Classification of Diseases (ICD) 10 code from the electronic medical record system (EMR-Epic). Patients referred for TFT tests for clinical suspicion of thyroid dysfunction, routine annual health assessments, or other medical evaluations were identified from the patient visit registry maintained in PHC, Dubai. The medical records of all selected patients were reviewed.

## Data collection

Demographic data, including age and gender, were collected from the EMR-Epic for all eligible patients. BMI was calculated using recorded height and weight from the medical records. Data on comorbidities, such as hypertension, dyslipidemia, type 1 diabetes, and ischemic heart disease, were also obtained from the medical records. These co-variates were selected due to their clinical relevance and potential association with hypothyroidism.

## Study population

**Fig. 1:**
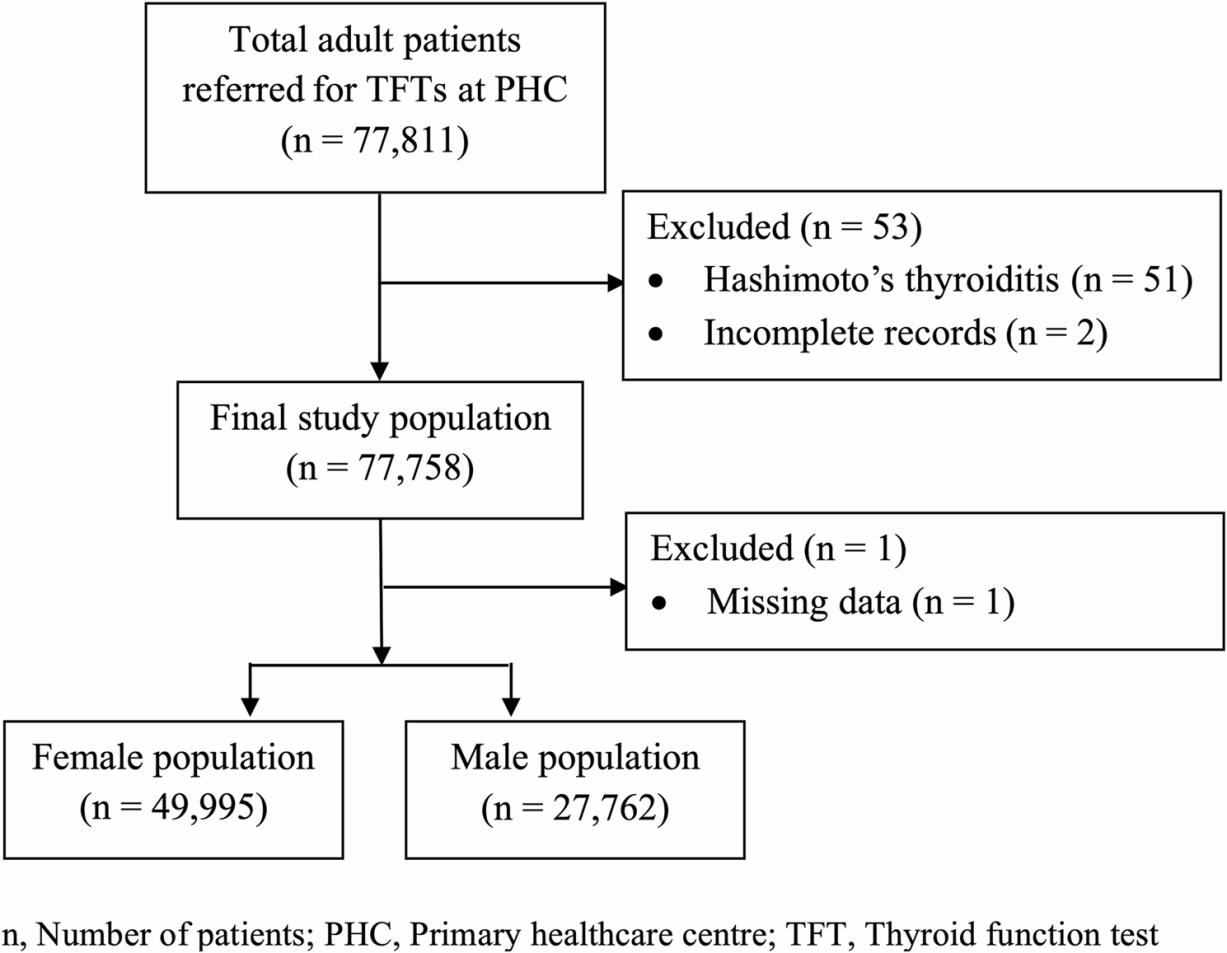
Patient selection for the study

The study population was identified based on the pre-defined inclusion and exclusion criteria. All patients ≥ 18 years having a medical file (Epic electronic health record of PHC visit at Dubai Health) with a TFT result and/or an ICD 10 code indicating hypothyroidism (ICD 10 code: E03.9, E06.3) or subclinical hypothyroidism (ICD 10 code: E03.9) were included in the study. Data on the autoimmune disease in patients was extracted based on ICD-10 coding in the medical record. Pregnant females were included in the study. Individuals < 18 years old were excluded. Patients with Hashimoto’s thyroiditis who were biochemically euthyroid and not on levothyroxine replacement therapy were excluded. All cases referred to as hypothyroidism in the text denote both overt hypothyroidism and subclinical hypothyroidism. A detailed summary of included and excluded individuals, along with reasons for exclusion, is presented in Fig. 1.

### Statistical analysis

Descriptive statistics and analyses were performed using the Statistical Package for the Social Sciences (SPSS, Version 23, IBM, Chicago, USA). The Shapiro–Wilk test was used to assess the normality of data distribution. Continuous variables with a non-normal distribution were presented as median with interquartile range (IQR) or range. Categorical variables such as hypothyroidism and obesity are presented as count (n) and percentage (%). The classification of obesity was done according to the standard WHO classification which includes BMI ranges as – underweight < 18.5, normal weight 18.5–24.9, overweight 25.0-29.9, obese ≥ 30 (class I – 30.0-34.9, class II – 35.0-39.9, class III > 40). The Chi-square test (χ^2^) and t-test were used to assess the association and differences between individuals with and without hypothyroidism. The difference in the prevalence of hypothyroidism among various age groups and gender categories was assessed using the χ^2^ test. The 95% confidence intervals (CIs) for prevalence estimates were calculated using the non-parametric methods in SPSS appropriate for proportion data. The *p-*value ≤ 0.05 was considered statistically significant. Adjusted analysis was not performed since the primary objective was to describe prevalence estimates of hypothyroidism.

## Results

### Demographic characteristics

A total of 77,811 adult patients (aged ≥ 18 years) attended the PHC for TFT from 1 st January 2018 to 23rd December 2020. The final study population (*n* = 77,758) comprised 64.3% females and 35.7% males. The median age of the patients enrolled was 42 years, with a median BMI of 28.41 (Table 1).

**Table 1:** General characteristics of individuals attending for thyroid function test

Demographics	Total	Without hypothyroidism	With hypothyroidism
Number of individuals, n (%)	77,758 (100)	76,112 (97.9)	1,646 (2.1)
Gender, n (%)			
Female	49,995 (64.29)	48,653 (63.9)	1,342 (81.5)
Male	27,762 (35.7)	27,458 (36.07)	304 (18.5)
Missing data	01 (0.001)	01 (0.001)	00
Median age in years (IQR)	42 (18–105)	42 (18–105)	41 (18–97)
Median weight in kg (IQR)	75.20 (65.00–87.30)	75.20 (65.00–87.40)	76.00 (66.00–86.83)
Median BMI (IQR)	28.41 (24.97–32.52)	28.40 (24.95–32.49)	29.36 (25.80–58.48)

*BMI* Body mass index, *IQR* Interquartile range

### Prevalence of thyroid dysfunction

Individuals without hypothyroidism comprised 97.9% (*n* = 76,112) of the total study population, with 63.9% being female and 36.07% male (Table 1). Their median age was 42 years, with a median BMI of 28.40. Detailed analysis revealed that the majority of them were in the age groups of 35–44 years (24%), 25–34 years (20%) and 45–54 years (19.8%). The remaining belonged to the age groups 55–64 (13.7%), < 25 (11.4%) and > 65 years (10.86%) (Table 2). The overall prevalence of hypothyroidism was 2.1% (*n* = 1,646 and 95% CI: 2.02–2.22%), consisting of 91.4% overt hypothyroidism and 8.6% subclinical hypothyroidism (Table 2). Among the patients with hypothyroidism, 81.5% were female and 18.5% were male (*p* = 0.000; χ^2^ test). Their median age was 41 years, having a median BMI of 29.36. A higher proportion of patients with hypothyroidism were aged 35–44 years (28.7%; 95% CI: 26.55–30.92%) followed by 25–34 years (22.7%; 95% CI: 20.64–24.68%), 45–54 years (20.0%; 95% CI: 18.11–21.98%) and 55–64 years (13.2%; 95% CI: 11.61–14.88%). Hypothyroidism was less common in extreme age groups (< 25 years at 5.8% and ≥ 65 years at 9.5%, *p* = 0.000) (Table 2). The highest prevalence was found in adults aged 35–44 years (2.52%; 95% CI: 2.30–2.75%), followed by 25–34 years (2.36%; 95% CI: 2.13–2.61%) and 45–54 years (2.14%; 95% CI: 1.91–2.37%). The prevalence in patients aged < 25 years was lowest (1.09%; 95% CI: 0.88–1.31%) (Table 3).

**Table 2:** Characteristics of the study population based on their age & BMI

Patient characteristics	Without hypothyroidism *n* (%) n (%) (*N*=76,112)		With hypothyroidism n (%)* (*N*=1,646)	
Hypothyroidism				
Overt	--------		1,504 (91.4)	
Subclinical	--------		142 (8.6)	
Age Group (in years)				
<25	8,686 (11.4)		96 (5.8)	
25–34	15,367 (20.2)		373 (22.7)	
35–44	18,260 (24)		473 (28.7)	
45–54	15,068 (19.8)		330 (20.0)	
55–64	10,461 (13.7)		218 (13.2)	
≥65	8,270 (10.86)		156 (9.5)	
Patients with available BMI records	74,235	95% CI (%)	1,613	95% CI (%)
Underweight	1,564 (2.1)	2.0–2.2	22 (1.3)	0.8–2.0
Normal weight	16,882 (22.7)	22.3–23.1	286 (17.7)	15.7–19.7
Overweight	26,213 (35.3)	34.8–35.7	547 (33.9)	31.6–36.2
Obese	29,576 (39.8)	39.4–40.3	758 (47)	44.6–49.5
Class I	17,547 (59.3)		459 (60.5)**	
Class II	7,622 (26)		192 (25.3)**	
Class III	4,280 (14.4)		98 (12.9)**	

The classification of obesity was done following the standard WHO classification of weight status which includes BMI ranges as: Underweight <18.5, Normal weight 18.5-24.9, Overweight 25.0-29.9, Obese ≥30 (Class I 30.0-34.9, Class II 35.0-39.9, Class III >40)

*(*p* = 0.000)

**(*p* = 0.606)

**Table 3:** Prevalence of hypothyroidism in individuals of different age groups

Age group (in years)	Total population (*N*)	Patients with hypothyroidism (*n*)	Prevalence rate (*n*/N*100) (%)	95% CI (%)
< 25	8782	96	1.09	0.88–1.31
25–34	15,740	373	2.36	2.13–2.61
35–44	18,733	473	2.52	2.30–2.75
45–54	15,398	330	2.14	1.91–2.37
55–64	10,679	218	2.04	1.77–2.31
≥ 65	8426	156	1.85	1.56–2.14

### Thyroid dysfunction and obesity

BMI records for 75,848 patients (without hypothyroidism: 74,235; with hypothyroidism: 1,613) were available. In patients with hypothyroidism (*n* = 1,613), obesity was prevalent in 47.0% (95% CI: 44.56–49.43%) and overweight in 33.9% (95% CI: 31.60–36.22%). However, in the non-hypothyroid group, obesity was prevalent in 39.8% and followed by overweight in 35.3% (Table 2). Among those categorised as obese, 59.3% belonged to class I, followed by 26% to class II and 14.4% to class III. Nearly half of the patients with hypothyroidism were obese (47%; 95% CI: 44.56%– 49.43%), followed by patients who were overweight (33.9%; 95% CI: 31.60–36.22%) (*p* = 0.000). Among patients with obesity, 60.5% (95% CI: 57.07%– 64.03%) had class I obesity followed by 25.3% (95% CI: 22.23–28.43%) with class II obesity and 12.9% (95% CI: 10.54%– 15.32%) with class III obesity (*p* = 0.606) (Table 2). Although the relationship between age and BMI was not linear, a trend of higher BMI in the older age group was evident.

### Associated comorbidities

In individuals without hypothyroidism, the prevalence of auto-immune disease was very low except for type 1 diabetes mellitus at 0.4% (Table 4). Other comorbidities included primary hypertension (5.26%), dyslipidemia (3.86%) and ischemic heart disease (0.39%). Among patients with hypothyroidism, 0.73% (95% CI: 0.33–1.13%) had type 1 diabetes mellitus and no co-diagnoses of other autoimmune diseases such as rheumatoid arthritis, systemic lupus erythematosus, pernicious anaemia, Addison’s disease, alopecia reported. However, a higher prevalence of dyslipidemia (8% and 95% CI: 6.71–9.33%), primary hypertension (6.5% and 95% CI: 5.31–7.69%) and ischemic heart disease (1% and 95% CI: 0.54–1.52%) was reported (Table 4).

**Table 4:** Frequency of co-morbidities in the study population

Comorbidities	Without hypothyroidism n (%) (*N* = 76,112)	95% CI (%)	With hypothyroidism *n* (%) (*N* = 1,646)	95% CI (%)
Autoimmune diseases				
Rheumatoid arthritis	27 (0.035)		0 (0)	
Systemic lupus erythematosus	22 (0.028)		0 (0)	
Type 1 diabetes	305 (0.40)	0.36–0.45	12 (0.73)	0.33–1.13
Alopecia areata	20 (0.026)		0 (0)	
Pernicious anemia	1 (0.001)		0 (0)	
Addison’s disease	0 (0)		0 (0)	
Vitiligo	37 (0.048)		1 (0.06)	
Other conditions				
Ischemic heart disease	302 (0.39)	0.35–0.43	17 (1)	0.53–1.47
Primary hypertension	4,006 (5.26)	5.10–5.42	107 (6.5)	5.29–7.71
Dyslipidemia	2,940 (3.86)	3.73–3.99	132 (8.01)	6.72–9.30

## Discussion

Thyroid hormone dysfunction, a common condition with potential health implications, is primarily treated with oral replacement of FT4 as levothyroxine, occasionally supplemented with intramuscular levothyroxine or oral liothyronine (FT3) [20]. Despite treatment availability and affordability, effectively controlling thyroid function and diagnosing the condition remains challenging, potentially leading to significant morbidity and mortality [21]. Therefore, understanding the prevalence and associated risks is crucial for effective treatment.

In our studied population, the overall prevalence of hypothyroidism was low (2.1%), with the majority of cases being overt hypothyroidism. Our data is comparable to the global average of 0.3%−5.3%, particularly reported in studies from Europe, North America and Australia [4]. However, our calculated prevalence of hypothyroidism in patients who visited the PHC for TFT screening is lower than similar studies conducted previously in the UAE which report prevalences of 8.8% [22] and 10.3% [23]. Furthermore, our finding is considerably less than the regionally reported prevalence, as seen in Bahrain (44%) [24], Saudi Arabia (29%) [25] and Jordan (26%) [26]. This discrepancy may be due to the fact that most of the studies were community-based and included both treated and untreated individuals, as well as those with overt and subclinical hypothyroidism. A large hospital-based study in Bahrain involving > 19,000 patients, reported a 44% prevalence of thyroid hormone dysfunction comprising treated euthyroid (15.1%), hypothyroid (20%) and subclinical hypothyroid (6.5%) cases [24]. Our focus on patients attending PHC starkly contrasts the data from Bahrain, likely because most patients in Bahrain were hospitalised and TFT may be less stable in an acute setting. Although the study from Saudi Arabia was in a PHC, the sample size was small, resulting in higher prevalence compared to our study, which included a larger patient cohort. Limited data exists on hypothyroidism prevalence in the MENA region. The Tehran thyroid cohort indicated annual incidence rates of 2 per 1000 for hypothyroidism and 7.62 per 1000 for subclinical hypothyroidism, with 6.7% progressing from euthyroid to thyroid dysfunction in an iodine-sufficient area of Iran [27]. A few studies from Saudi Arabia have looked at general prevalence in adults within the PHC setting [25, 28].

Our data strongly indicate the preponderance of hypothyroidism among women similar to most of the global and regional study findings [12, 13, 26]. A study from Jordan reported a high prevalence of overt and subclinical hypothyroidism (17.2% in females, 9.1% in males) among 7085 patients [26]. Interestingly, an increased prevalence of hypothyroidism was not observed in older age groups, aligning with results from Bahrain [24]. The highest prevalence was found in the middle age group (34–44 years), followed by the age group 25–34 years, whereas in the age group more than 50 years the rate was lower in our study. Conversely, contrasting results were reported in Saudi Arabia, where a higher rate of hypothyroidism was reported among individuals aged more than 50 years, possibly due to the use of a low cut-off TSH value of 4.2 mIU/L [24, 25]. Subclinical hypothyroidism was only entered as an ICD-code in a small percentage of patients, around 8.6% of those confirmed to have hypothyroidism. A study in a community healthcare centre in Saudi Arabia reported a prevalence of subclinical hypothyroidism in the general population at 10.3%, reflecting a prevalence similar to international values [4, 23].

In our study, the number of obese individuals with hypothyroidism was higher (47%) than those without hypothyroidism (39%). Additionally, a majority of patients with thyroid dysfunction were obese, revealing a significant association between hypothyroidism and obesity, although the association with obesity classes was not statistically significant. The prevalence rates of hypothyroidism were comparable to obesity rates in patients with type 2 diabetes mellitus in a study conducted in the UAE [29–31]. These data also raise concerns about the risk of the development of cardio-metabolic disease in patients with hypothyroidism as obesity is associated with cardiometabolic conditions. Thyroid dysfunction has been known to be associated with certain complications, like cardiovascular disease, type 1 diabetes in children and chronic kidney disease [32–34]. In the GCC, studies on hypothyroidism often focused on its association with other conditions, such as autoimmune disorders, type 2 diabetes mellitus, pregnancy, Down’s syndrome, or congenital disease [35–42]. Additionally, studies have demonstrated an increased incidence of hypothyroidism with type 2 diabetes mellitus [43]. Consistent with this, in our study, patients with hypothyroidism exhibited a higher incidence of type 1 diabetes mellitus, along with dyslipidemia, primary hypertension and ischemic heart disease. Individuals without hypothyroidism had a lower prevalence of autoimmune diseases like type 1 diabetes and vitiligo. These increased rates of co-morbidities in patients with hypothyroidism indicate that cardiometabolic risk is higher in such patients than in the general population.

This study has several strengths that contribute to its significance. Firstly, considering that Dubai Health provides services to the majority of the population with most patients being Emirati, our study can be considered a good representation of the local population. Moreover, it leverages a large sample size, providing robust statistical power. The unique focus on patients visiting the PHC who are referred for TFTs distinguishes this study by targeting individuals less susceptible to acute illnesses than those typically in hospital settings. Additionally, the study uses TFT to ascertain control and medication records. Despite providing valuable insights, it is essential to acknowledge certain limitations inherent in the study. Our study was not a population-based screening assessment with sample not randomly included regardless of symptomatology. It only enrolled patients who were referred for TFTs at the PHC, which largely limits the generalisability of these findings to a broader population. Furthermore, information regarding the number of patients enrolled in Dubai Health during study period and the proportion of overall population of Dubai undergoing checkup at Dubai Health the population were not available in our dataset. Also, the retrospective nature of research design poses a constraint, potentially impacting the accuracy of findings. The reliance on data extraction through ICD-10 codes may not reflect accurate diagnoses. Furthermore, the study could not differentiate between subclinical and overt hypothyroidism owing to incomplete documentation on thyroid antibody status.

## Conclusions

Hypothyroidism is prevalent among patients visiting PHC in Dubai with a low prevalence, similar to those reported in Europe and North America and less than those in the neighbouring Gulf countries. It is most prevalent among young and middle-aged individuals, particularly women and is associated with higher rates of obesity and cardiometabolic disease. Healthcare providers should be aware of the associated risks and screen patients accordingly.

## Data Availability

The datasets used and/or analysed during the current study are available from the corresponding author on reasonable request.

## Abbreviations

χ^2^: Chi-square test
BMI: Body mass index
FT3: Free T3 or Triiodothyronine
FT4: Free T4 or Thyroxine
GCC: Gulf Cooperation Council
ICD: International Classification of Diseases
MENA: Middle East and North Africa
PHC: Primary Healthcare Centers
SD: Standard deviation
SPSS: Statistical Package for the Social Sciences
TFT: Thyroid function test
TSH: Thyroid-stimulating hormone
UAE: United Arab Emirates
